# Royal Sussex Jennerian Institution

**Published:** 1805-10-01

**Authors:** 


### ROYAL SUSSEX JENNERIAN INSTITUTION.

*Lewes, Aug.* 13, 1805.

AT a Meeting of Directors of this Society, held here this day, present,

The EARL OF CHICHESTER, President.

J. FULLER, Esq. M. P.    J. THOMAS, Esq.

T. PARTINGTON, Esq.    H. SHELLEY, Esq. M. P.

G. SHIFFNER, Esq.

the following report of the Medical Council was read:—

*Annual Report of the Medical Council.*

The list of patients inoculated for cow-pock, from July 1, 1804, to July 1, 1805, at Chichester, Arundel, Midhurst, Horsham, Brightelmston, Lewes, Seaford, Eastbourn, and Battle, by the members of this institution, amount to 946, of which number 509 persons were inoculated gratuitously. No lists were received from the other stations.

With the report of inoculations for cow-pock during last year, the Medical Council beg leave to represent to the Board of Directors, that nothing has occurred in the practice of any member of this society, (as far as they have been able to collect) which could shake their confidence in the efficacy of cow-pock, as a preventative of small-pox.

The Medical Council wish also to state, that the present list of inoculations for cow-pock in this county, during last year, is less than it otherwise would have been, for the following reasons:

1. The number of persons inoculated, previous to the institution of this society, left in many parts of the county few subjects for inoculation. For from particular enquiry, it appears that the practice of vaccine inoculation commenced in Sussex, as early as March 1799, (a few months after Dr. Jenner's first publication of his discovery,) and that upwards of ten thousand persons had been inoculated previous to July 1804, exclusive of inoculations among the military.

2. A

2. A number of individuals have been inoculated for cow-pock, by persons not medical.

It is to be feared that some of those so inoculated may not have had genuine cow-pock, and therefore may at a future time catch small-pox; by which they will not only suffer themselves, but bring discredit on vaccine inoculation.

3. Many are convinced of the efficacy of cow-pock inoculation, who from indolence and procrastination, defer having recourse to it till they hear of small-pox breaking out among their neighbours.

4. There are still not a few who from ignorance, doubt, or prejudice, refuse to inoculate their children with cow-pock. Some are even averse to inoculation altogether.

The Medical Council are of opinion that it would greatly promote the object of this society, if gentlemen would use their influence to have the parish poor inoculated quarterly by their respective parish surgeons.

For though the Medical Members of this institution are sincerely desirous to promote vaccine inoculation among all classes, and are ready to inoculate at the different stations the children of the poor gratuitously, yet they have not thought it expedient to extend their gratuitous inoculation to such paupers as are actually chargeable to their parishes.

The Medical Council cannot conclude this Report without noticing to the Board of Directors, the assistance they have received from the Royal Jennerian Society of London, by the ready supply of cow-pock matter, to any Medical Gentleman of this county who applied for it, which happened on several occasions.

(Signed) THO. BLAIR,
President of the Medical Council.

Wm. Brewster, Secretary.

---

The Board of Directors having heard the above Report read, passed the following resolutions:

Resolved, It appears to this Board that the proposal of the Medical Council, respecting quarterly inoculation in the different parishes would be attended with the best effects. The Directors accordingly recommend this to the attention of all parish officers; and they most earnestly request the assistance and influence of the Clergy in promoting so desirable an object.

Resolved,

Resolved, That the thanks of this Board be given to the Medical Council, and the other medical members, for the active part they have taken in furthering the views, and promoting the advantages to be derived from this institution.

(Signed) CHICHESTER, President.

Subscriptions are received at the Lewes Bank, by Mr. Whitfield, Treasurer.

A donation of five guineas, or an annual subscription of one guinea, constitutes a governor.

